# Early events in TCR signaling – the evolving role of ITAMs

**DOI:** 10.3389/fimmu.2025.1563049

**Published:** 2025-04-24

**Authors:** Paul E. Love, Teri Hatzihristidis, Jayna Bryant, Guillaume Gaud

**Affiliations:** Section on Hematopoiesis and Lymphocyte Biology, Eunice Kennedy Shriver, National Institute of Child Health and Human Development, National Institutes of Health, Bethesda, MD, United States

**Keywords:** T cell receptor, ITAM, signaling, ligand discrimination, antagonism, model

## Abstract

The T cell antigen receptor (TCR), a multiprotein complex essential for adaptive immunity, is composed of variable TCRα and TCRβ subunits responsible for antigen recognition and six invariant signal transducing CD3 and ζ subunits. Peptide-MHC (pMHC) binding by TCRα/β dimers results in the transmission of signals mediated by the CD3 and ζ subunits, which each contain one or more immunoreceptor tyrosine-based activation motifs (ITAMs). Several other immune receptors utilize ITAMs for signal transduction; however, while each of these receptors includes between one and three signaling subunits with a single ITAM, the TCR is strikingly unique, containing a total of ten ITAMs distributed within the six CD3 and ζ subunits. Numerous studies conducted over the past twenty-five years have attempted to determine the purpose for the structural singularity of the TCR. From these investigations, three models of ITAM function have emerged: signal discrimination (selective effector binding to different ITAMs), signal amplification (additive effect of ITAMs) and signal duality (activation and inhibition by ITAMs depending on context). In this review, we revisit the long history of ITAM research, which despite intensive investigation, has yet to provide a clear consensus for the role of TCR signaling subunit and ITAM multiplicity. We conclude by relating results from our recent study of the three tandem ζ ITAMs that suggest that at least some TCR ITAMs can transmit both activating (amplifying) and inhibitory signals depending on the affinity of the pMHC-TCR interaction and the subunit context, contributing to and enabling the nuanced regulation of T cell responses by the TCR and helping explain the exquisite ligand discrimination displayed by the TCR. These findings also suggest a model for ligand-mediated antagonism, a well-documented but poorly understood atypical TCR signaling response. Finally, we examine the implications of these findings which provide the basis for a new functional model for TCR ITAM multiplicity. A comprehensive understanding of the roles of ITAMs and the CD3 interactome will emerge from continued investigation, shedding light on the fascinating but still incompletely understood most proximal steps in the TCR signaling cascade.

## Overview of ITAMs and TCR signaling

**Figure 1:**
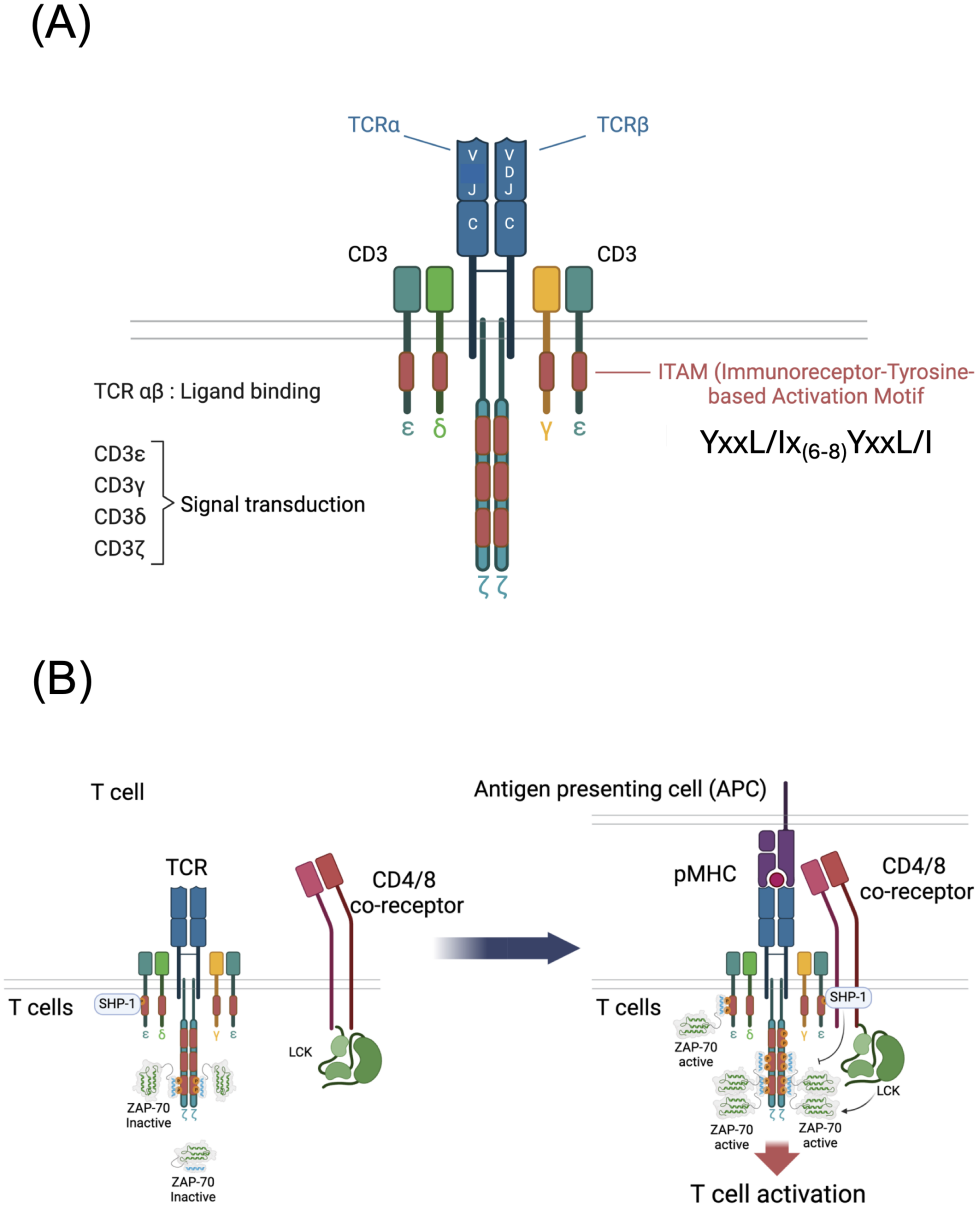
Structure and function of the T cell antigen receptor (TCR). **(A)** Subunit structure of the TCR showing the antigen binding TCR α and β chains and the signal transducing CD3γ, δ, ε and ζ chains. The TCRβ chain is generated by V-[D]-J recombination and the TCRα chain is generated by V-J recombination. **(B)** Schematic of early TCR signaling showing resting and ligand engaged T cells. In resting T cells that bind self-peptide + MHC (pMHC), di-phosphorylated ζ chain ITAM tyrosines recruit an inactivated form of ZAP-70 and mono-phosphorylated ζ chain ITAM tyrosines recruit the tyrosine phosphatase SHP-1. Following TCR engagement by antigen pMHC presented by APCs, CD4 or CD8 binding of MHC brings the tyrosine kinase LCK to the vicinity of the TCR resulting in phosphorylation of ζ and CD3 subunit ITAM tyrosines that recruit ZAP-70 tyrosine kinase. LCK and ZAP-70 phosphorylate and activate ZAP-70 triggering downstream signaling. Mono-phosphorylated ITAMs if present may recruit the inhibitory phosphatase SHP-1 which is capable of inactivating ZAP-70.

The subunit composition of the TCR complex was resolved in the mid-1980s from studies reported by several laboratories (1–3). The current consensus structure of the TCR is a multimer with the following configuration: TCRα, β, CD3γε, CD3δε, ζζ (**Figure 1A**) although dynamic changes in TCR stoichiometry and/or conformation have been proposed (2, 4). The TCR subunits exhibit a clear dichotomy of function, with the clonotypic TCRα and TCRβ chains, derived from V-[D]-J recombination, solely responsible for antigen recognition and the invariant CD3γ, δ, ε and ζ chains solely responsible for signal transduction (**Figure 1A**). In 1989, Michael Reth identified in each CD3 and ζ chain as well as in several other immunoreceptor subunits, a semi-conserved di-tyrosine amino acid sequence, subsequently designated an Immuno-receptor Tyrosine based Activation Motif (ITAM) with the consensus: YxxL/I-x_6-8_-YxxL/I where Y=tyrosine, L=leucine, I=isoleucine and x=any amino acid (5). Interestingly, whereas all other ITAM containing immune receptor subunits including CD3γ, CD3δ and CD3ε contain a single ITAM, ζ contains three ITAMs (**Figure 1A**).

**Figure 2:**
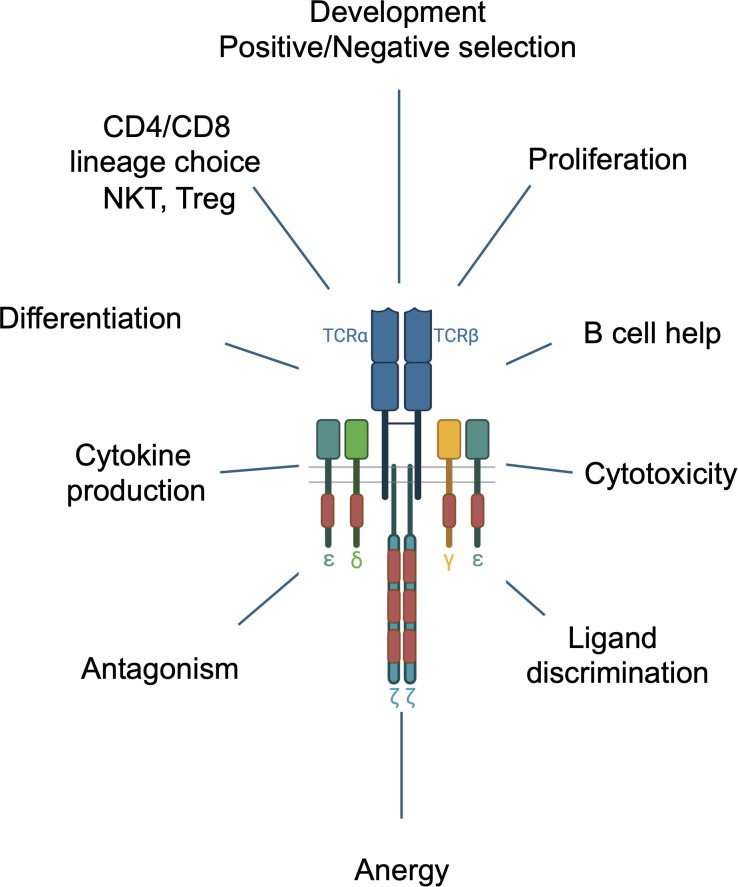
Multiple functions of the TCR during T cell development and in mature T cells.

The precise steps of TCR signal initiation remain incompletely understood although several models have been proposed and are reviewed here (6, 7). A popular and experimentally supported model posits that co-engagement of peptide-MHC (pMHC) by the TCR and a conserved region of MHC by CD4 or CD8 co-receptor results in recruitment of co-receptor bound SRC-family tyrosine kinase LCK to the proximity of ITAMs leading to the phosphorylation of ITAM tyrosines by LCK (8) (**Figure 1B**). Sufficient TCR-pMHC dwell time results in the tyrosine phosphorylation (pY) of the tyrosines of both YxxL/I motifs within an ITAM (i.e., pYxxL/I-x_6-8_-pYxxL/I) creating a high affinity binding site for the dual SH2 domain hematopoietic lineage specific tyrosine kinase ZAP-70 (9). ITAM-recruited ZAP-70, which is tyrosine phosphorylated and activated by LCK and by autophosphorylation, then diffuses into the cytoplasm in the vicinity of the TCR where it binds to and tyrosine phosphorylates several target molecules including, most notably, the transmembrane scaffolding adapter protein LAT (10). These critical early steps in TCR signaling result in the subsequent triggering of multiple downstream signaling pathways including those that activate MAP kinases, protein kinase C, and Vav, and induce calcium release from intracellular stores, resulting in activation of the transcription factors Fos, Jun, AP-1, NF-κB and NFAT and leading to proliferation, cytokine production and various lineage specific T cell effector functions (**Figure 2**) (10).

Most ITAM tyrosines within the CD3 chains (specifically, the CD3γ, CD3δ, and CD3ε ITAM tyrosines and the first ζ ITAM tyrosines) are unphosphorylated in naïve, unstimulated T cells (11); however, it has been known for decades that at least some of the ITAM tyrosines within ζ are phosphorylated in *ex vivo* mouse (12–14) and human (15) thymocytes and resting peripheral T cells. The term ‘resting’ in this case refers to cells that are not stimulated by activating ligands but are nevertheless engaged by self-ligands *in vivo* which are not inert but instead are thought to initiate transduction of low intensity, homeostatic signals through the TCR that are important for T cell survival (16). This concept was confirmed by the finding that removal of thymocytes or T cells from *in vivo* cell-cell interactions that present self-ligands to the TCR for several hours results in the complete de-phosphorylation of ζ (14, 17). The mass of unphosphorylated ζ is 16 kDa. Partially tyrosine phosphorylated forms of ζ range from 21 kDa to <23 kDa with a predominant p21 form, and ζ that is completely tyrosine phosphorylated (phosphorylation of all six ITAM tyrosines) has a mass of 23 kDa (11).

A particularly intriguing early finding was that, in both thymocytes and peripheral T cells, p21ζ is associated with ZAP-70, and in turn, the stability of phosphorylated p21ζ is dependent upon ZAP-70 binding (11, 18, 19). However, this ‘constitutively’ ζ-associated ZAP-70 was found to be auto-inhibited and therefore catalytically inactive (11, 13, 19). Antibody or pMHC TCR stimulation results in the generation of fully phosphorylated p23ζ and the recruitment, phosphorylation and activation of ZAP-70 (11). The ITAM tyrosine phosphorylation pattern of p21ζ was eventually resolved in 2000 by Van Oers and colleagues who found that both tyrosines in the second and third ITAM of p21ζ are phosphorylated and both of these ITAMs are thought to be complexed with inactive ZAP-70 (**Figure 1B**) (11, 20). In addition, as discussed below, recent data have shown that the tyrosine phosphatase SHP-1 (encoded by *Ptpn6*) is associated with both un-stimulated and stimulated TCRs (**Figure 1B**) (21). As mentioned above, TCR engagement results in the phosphorylation of all six ζ ITAM tyrosines, generating p23ζ and the recruitment and activation of ZAP-70 (**Figure 1B**) (11). A study by Allen and colleagues, using antisera specific for individual ζ-ITAM tyrosines, suggests that fully activating pMHC/TCR interactions result in phosphorylation of the first then the second tyrosine within the membrane proximal ITAM of ζ (22).

The mechanism(s) responsible for initiating and regulating tonic ζ ITAM phosphorylation have not been precisely defined. A number of models have been proposed including those that involve lipid mediated sequestration to the inner plasma membrane at rest followed by activation induced conformational change in the case of CD3ε and ζ, or the involvement of active cytosolic LCK and inhibitory tyrosine phosphatases (e.g., CD45) whose functionality is determined by molecular segregation upon activation (6, 23). However, there has been much debate about these models and the reason/purpose for basal ζ phosphorylation still remains unclear. With this in mind, Malissen and Bongrand proposed the ‘warm-up’ model of TCR triggering which posits that pre-assembled complexes consisting of the CD3 and/or ζ chains and ZAP-70 exist to ‘prime’ resting T-cells for activation upon ligation with foreign pMHC (6). However, the results obtained with inactivated 6Fζ discussed below instead suggest a potential inhibitory function for ζ and perhaps also ZAP-70/ζ complexes in resting T cells (21). Constitutively active LCK is detected in 40% of ‘resting’ T cells, and at the basal state, the TCR is regularly interacting with self-pMHC to trigger partial phosphorylation of ζ ITAMs and association of ζ with catalytically inactive auto-inhibited ZAP-70 (23–25). What has remained unresolved, and will become a recurrent issue in this review, is the function, if any, of p21ζ and p21ζ−associated ZAP-70 in pMHC induced T cell activation.

## The multi-ITAM conundrum

A striking and singular feature of the TCR is its large number of signaling subunits and ITAMs compared with all other ITAM containing immune receptors, including the B cell receptor (BCR), several Fc receptors, Ly49 and TREM-1 & -2 which contain one, two or a maximum of three signaling subunits and ITAMs. Since the discovery of the TCR subunit structure, a key question has been why so many signaling subunits and ITAMs? What does the TCR do that requires such a unique subunit configuration and an unusually high ITAM multiplicity? A possible explanation may lie in the fact that the TCR controls and regulates an unusually wide range of cellular responses including activation, proliferation, cellular specialization, apoptosis, and B cell help, distinguishing it from the aforementioned more functionally restricted ITAM containing receptors (**Figure 2**). The TCR also exhibits a remarkable capacity for ligand discrimination, capable of responding to ligands that bind with a broad range of affinities (1-100μM) with graded signaling responses. Moreover, the TCR controls multiple distinct developmental events including positive and negative thymocyte selection and lineage choice (CD4, CD8, Treg) in the thymus, and differentiation of mature T cells into specialized subsets (e.g., CD4+ T cells into Th1, Th2, Th17, Tregs) as well as regulating lineage-specific functions including suppression (Tregs), cytokine production (CD4+ T cells) and cellular cytotoxicity (CD8+ T cells) (**Figure 2**). Each of these functions, which are also controlled by additional signals provided by co-receptors and cytokines, is tightly regulated by TCR signaling pathways ensuring the induction of the appropriate immune responses for defense against pathogens, cancer cells, immune surveillance and self-tolerance. Finally, the TCR is capable of responding to different ligands with different signaling outputs that can initiate distinct outcomes including antagonism, anergy and memory cell formation and can participate in receptor cross regulation/cross-talk (26, 27) (**Figure 2**).

## Functional models for TCR ITAM multiplicity

Although T cell multifunctionality and plasticity may serve as a compelling justification for the complexity of the TCR, as will be discussed next, experimental data identifying or supporting a specific mechanism for TCR ITAM multiplicity has been frustratingly lacking, and indeed, the models for ITAM multiplicity have not changed substantially since they were first proposed over twenty-five years ago (28).

### Discrimination

A particularly attractive hypothesis that emerged shortly after the discovery of ITAMs was the idea that individual ITAMs may recruit different binding partners or exhibit different binding affinities for the same partners since ITAM sequences are only semi-conserved (amino acids other than the two tyrosine residues are variable in different ITAMs) (5, 29). Co-immunoprecipitation experiments, which used synthetic ITAM phospho-peptides as bait, reported preferential binding or variable binding strength of several key effectors (ZAP-70, Shc, Grb2, Fyn, RasGAP, p85/PI3Kinase) to a subset of TCR ITAMs, although, in most cases, not all TCR ITAMs were tested and in no cases was any protein found to bind only a single ITAM (30–33). It is important to note that these studies only assessed recruitment of potential effector proteins to ITAMs. Selective recruitment of Nck, p85 and acidic phospholipids has been attributed to non-ITAM sequences within CD3ε (34–36) and ζ has been shown to contain a GTP binding motif (37); however, these non-ITAM interactions are not discussed in this review. A second approach tested the signaling potential of chimeric proteins that fused unrelated extracellular domains that could be antibody cross-linked (e.g., CD8, CD25) to the intracellular domains of individual TCR signaling subunits, typically CD3ε or ζ. The consensus result was that individual ITAMs, or the CD3 or ζ cytoplasmic domains were capable of delivering signaling responses that were qualitatively similar to those of the intact TCR complex (38–40) although one study reported that signaling by the ζ and CD3ε cytoplasmic domains generated a different set of phosphoproteins (41), and a second reported that CD3ε but not ζ induced [Ca^++^] mobilization (42). Unfortunately, these early studies have not been confirmed or followed up by additional experiments. Arguably more relevant are studies that evaluated the signaling responses of intact multi-subunit TCRs expressing truncated (ITAM deleted) signaling subunits or point mutations of CD3 or ζ chain ITAM tyrosines. Two groups in addition to our own generated TCRs that contain ζ subunits with ITAM deletions or tyrosine to phenylalanine mutations of ITAM tyrosines (43–49). In the cases where mutagenesis was performed with transgenes or in mouse embryonic stem cells (43–45, 47, 48), TCR-mediated *in vivo* functions including thymocyte selection, effector responses and T cell homeostasis could be evaluated. Remarkably, all of these studies found that ζ ITAM-mediated signals were not required for T cell development if the CD3 subunit ITAMs were preserved. In similar experiments, the CD3γ ITAM (50) and CD3δ ITAM (51) were found to be dispensable for thymocyte maturation. One exception to these consensus results was a report using a retrogenic experimental approach that concluded that ITAM diversity is necessary for normal T cell development (52), perhaps because differences in ZAP-70 binding affinity are necessary for providing TCR sensitivity and ligand discrimination (53). However, the generation of p21ζ and the assembly of ZAP-70/ζ complexes in resting and activated *ex vivo* thymocytes and T cells was not examined in that study (52). Studies analyzing the binding of potential effector proteins including ZAP-70, Shc, PI3K(p85), Grb2, Fyn, and Ras-GAP to TCR ITAMs using synthetic phosphorylated peptides have shown preferential and sometimes selective binding of effectors to specific TCR ITAMs. Only one study compared binding to all six TCR ITAM sequences (ζ1, ζ 2, ζ 3, CD3-γ, -δ, and -ε), highlighting selective ITAM interactions particularly with Shc, p85, Grb2, and Fyn. However, these findings remain preliminary, as follow-up studies are lacking, and it is unclear if the observed binding patterns *in vitro* accurately reflect *in vivo* interactions within the intact TCR complex, where cooperative or ordered effector binding may occur (54).

### Evidence for ITAM discrimination in CAR signaling

The role of individual ITAMs in Chimeric Antigen Receptor (CAR)-mediated T cell activation, investigated by modification of the 3 ITAMs within the ζ cytoplasmic domain, has revealed distinct contributions of individual ITAMs to CAR signal strength and to CAR T persistence and therapeutic efficacy. In a significant advance for CAR T cell design, Sadelain’s group explored the effect of different configurations of ζ ITAMs on therapeutic efficacy (55). By systematically mutating tyrosine residues across the three ζ ITAMs (1,2,3), the study revealed that a single functional ITAM at the proximal position (1XX) outperformed the conventional ζ(1,2,3) ITAM CAR design *in vivo*, in CD28/ζ CARs despite similar short-term *in vitro* functionality. This optimized 1XX configuration achieved complete tumor eradication and enhanced survival in pre-B acute lymphoblastic leukemia models, while promoting increased T cell persistence, delayed differentiation, and reduced exhaustion. Transcriptomic analyses confirmed that 1XX CAR T-cells display a balance in effector and memory programs, resembling stem cell memory T cells more closely than conventional ζ (1,2,3) ITAM CARs. In contrast, the XX3 variant with only the distal ζITAM active showed strong memory features but insufficient anti-tumor activity compared to control ζ (<ξ>1,2,3) ITAM CARs, demonstrating the importance of both ITAM dosage and positioning. This work establishes that CAR ITAM calibration can optimize the delicate balance between T cell differentiation and effector function, a finding with immediate translational potential.

In a recent study, Majumdar et al., designed second generation murine anti-CD19 CD28/ζ based CARs containing three copies of only one of the three ζ ITAMs (referred to as ζ111, ζ222 and ζ333) (56). ζ111 CAR T cells exhibited stronger activation, as evidenced by faster degranulation and higher levels of effector cytokines (IFNγ and TNFα). Despite similar cytotoxicity across all CAR variants, ζ111 CAR T cells were less abundant after tumor killing, indicating they may be more prone to activation-induced cell death due to higher CAR signal strength. In contrast, ζ333 CAR T cells displayed a weaker activation profile, which correlated with lower expression of exhaustion markers compared to ζ111 or ζ222 CAR T cells. The study also revealed distinct signaling profiles: ζ111 CAR T cells showed stronger phosphorylation and activation of ITAM-proximal signaling molecules (ZAP-70, LAT, PLCγ), resulting in higher calcium (Ca2+) signaling, while ζ333 CAR T cells exhibited weaker signaling and a lower Ca2+ response. The results suggest that ζ111 CAR T cells have overall stronger signaling, while ζ333 CAR T cells exhibit weaker signaling, potentially reducing exhaustion. The authors hypothesize that these differences in signaling are due to variations in phosphorylation kinetics and binding preferences of the SRC family kinases (LCK and FYN) and the phosphatase CD45, influencing ZAP-70 recruitment and activation. These findings and those of the Sadelain group (55) suggest that ITAM engineering represents a promising approach for fine-tuning CAR T cell functionality. However, the translatability of results obtained with CARs to the multisubunit TCR, if any, remains unclear.

### Amplification

This model of ITAM function extends from the logical assumption that although there may be a signaling threshold for individual effector responses, in most cases, given the shared ZAP-70 binding capability of all ITAMs, di-phosphorylation of increasing numbers of TCR ITAMs results is expected to result in an additive effect on the signaling response through the recruitment of increasing numbers of ZAP-70 molecules to the TCR complex. Indeed, most experimental data have supported this supposition, particularly with regard to *in vivo* signaling dependent cellular events where the effects of TCR ITAM reduction were analyzed in the context of TCR pMHC binding affinity. Several studies have demonstrated that the CD3 (γ, δ, ε) subunits are capable of transducing signals required for T cell development and T cell effector functions in the absence of ζ mediated signals (40, 43–46). In studies using different experimental models of ζ ITAM reduction, mice expressing TCRs with inactivation or deletion of ζ ITAMs displayed no or only minor defects in the development of polyclonal T cells (20, 43, 48, 57–59) although some signaling and effector functions exhibited a quantitative dependence on ζ ITAM numbers (45, 57–61). Two studies reported that below a critical threshold (i.e., deletion of all ζ ITAMs and one or more of the CD3γ, δ, or ε ITAMs) T cell development is arrested at the immature CD4^-^CD8^-^ DN3 stage where pre-TCR signals are required for subsequent development (51, 57). Only when the TCR specificity and the positively selecting ligand affinity was fixed by expression of a transgenic TCR were clear defects in later development (positive selection and negative selection) observed by removal of the ζ ITAMs, and importantly, the severity of the developmental defect correlated inversely with the known or presumed affinity of the positively selecting TCR/pMHC interaction (44, 45, 57, 58, 62–64).

Interestingly, the generation of T cells requiring high-affinity interactions for their development, such as T follicular helper (Tfh) T cells or invariant natural killer T (iNKT) cells, is impaired in mouse models lacking functional ζ ITAMs (59, 65). This observation suggests that ζ (and perhaps by extension, a full complement of TCR ITAMs) is essential for the development of these cell types, which rely on strong TCR signaling during their maturation. Similarly, our recent study indicates that ζ ITAMs are crucial for robust TCR signaling in response to antigen in peripheral αβ T cells, facilitating effective immune responses, but are not essential for responses initiated by lower affinity TCR interactions (21). These findings align with the notion that ζ ITAMs are vital for amplifying signals from high-affinity interactions and for functional activation of peripheral T cells. This understanding may appear to contrast with the observed defects in thymocyte positive selection of ζ-ITAM deficient mice described earlier, where positive selection primarily depends on low-affinity interactions with self-major histocompatibility complex (MHC) molecules. This discrepancy implies that the role of ζ ITAMs may vary depending on the developmental stage and activation context of αβ T cells. A plausible explanation for these differences is that thymocytes and peripheral αβ T cells exhibit distinct TCR signaling thresholds, in part due to different expression levels of key signaling molecules, including THEMIS, TESPA, PTPN22 and PTPN6 (SHP-1) (10). Thymocytes, tuned for positive selection, have a lower TCR signaling threshold to permit survival and maturation following weak interactions with self-MHC. In contrast, peripheral αβ T cells, which are not activated by positively selecting ligands, require stronger, sustained signals for activation, making them more dependent on ζ-ITAM mediated signal amplification. Variations in the expression levels of signaling proteins between thymocytes and peripheral T cells may further contribute to the divergent signaling requirements observed in these populations.

A more recent study where the signaling effects of increased numbers of TCR ITAMs was analyzed with *in vitro* assays concluded that multiple TCR ITAMs increased the probability of activation following ligand engagement rather than amplifying signal output per se (66). A systems model of TCR proximal signaling concluded that the multi-subunit/multi-ITAM TCR configuration enabled signal amplification, ultra-potency and ultra-sensitivity (53). In summary, considerable experimental data support the idea that, while perhaps not universal, at least some signaling responses and developmental stages are sensitive to reduction in total TCR ITAM numbers regardless of the specific ITAM deleted.

### ITAM signaling duality

The concept of dual activating and inhibitory ITAM signaling has been well established for immune receptors other than the TCR including Fc receptors, the B cell receptor, and TREM-1,2 (67–69). Inhibitory ITAMs are generated by low affinity ligand interactions of ITAM subunit-associated receptors that result in phosphorylation of a single ITAM tyrosine (N or C tyrosine but not both) leading to recruitment of the inhibitory SH2 domain phosphatases SHP-1 or SHIP rather than recruitment of activating SYK family kinases (67–69). Thus, the premise of inhibitory ITAMs is that an ITAM that delivers activating signals by recruiting a dual SH2 domain SYK family kinase when both tyrosines within the ITAM are phosphorylated following relatively high affinity receptor ligation can also be mono-phosphorylated following low affinity receptor engagement leading to recruitment of a single SH2 domain inhibitory phosphatase. Despite considerable interest in demonstrating inhibitory ITAM signaling in the context of the TCR, direct evidence has been frustratingly lacking. A 1999 study by Kersh et al. performed with 3.L2 hybridoma cells demonstrated that co-engagement of the TCR with some ITAM Y/F mutant CD8-ζ chimeras that could only be partially phosphorylated resulted in inhibition of TCR-mediated IL-2 production (70). Interestingly, co-engagement of one of the Y/F CD8-ζ chimeric constructs inhibited T cell proliferation in transgenic mice; however; a mechanism for the inhibitory effect, specifically involvement of an inhibitory phosphatase, was not identified in this study (70). Moreover, an ‘inhibitory ITAM’ effect was not demonstrated in the context of the TCR. In a complementary approach, using Jurkat T cells expressing phospho-mimetic CD3ε ITAMS, a recent study described an inhibitory effect of irreversible ITAM phosphorylation on T cell activation, ascribed to altered coordination of LCK recruitment despite increased ZAP-70 binding, perhaps providing some insight into how ITAM phosphorylation kinetics and ZAP-70 can contribute to ITAM signaling duality (71). Nevertheless, historically, the search for an inhibitory function for TCR ITAMs has mainly been associated with experiments designed to understand the phenomenon of TCR antagonism.

### TCR antagonism

The concept of TCR antagonism has been extensively investigated since it was discovered that certain pathogen-derived altered peptide ligands (APLs) can suppress T cell activation as a strategy for immune evasion (72, 73). Since its discovery in the early 1990’s, TCR antagonism [reviewed in (74)] sparked the search for mechanisms for inhibitory TCR signaling to explain this phenomenon. A key early finding was that TCR stimulation by agonist and antagonist ligands resulted in different ζ phosphorylation patterns. Specifically, TCR stimulation by agonists causes the generation of approximately equal amounts of p21ζ and p23ζ whereas stimulation with antagonist ligands leads to the generation of predominantly p21ζ with very little generation of p23ζ (22, 75–78). Notably, agonist but not antagonist stimulation results in the activation of ZAP-70 (75, 76). These findings established a correlation between distinct ζ phosphorylation patterns and either abnormal (partial or ineffective) T cell activation (antagonism) or full T cell activation. However, since those early observations, a clear mechanism for antagonism linked to the dominant p21ζ phosphorylation pattern has not emerged. Instead, a plethora of publications have related diverse and often conflicting findings regarding TCR antagonism. For example, some studies (76, 77) but not others (44, 64, 79) have correlated antagonism with incomplete or altered ζ ITAM phosphorylation. In some studies, p21ζ was speculated to function as an inhibitory ITAM capable of recruiting SHP-1 (67); however, others have suggested that p21ζ represents a primed state that facilitates rapid ZAP-70 recruitment and T cell activation after TCR engagement (17). There have also been conflicting results concerning whether antagonist pMHC/TCR interactions generate ‘inhibitory’ TCR signals that can act in trans meaning that the inhibitory signal generated at one TCR can diffuse to inhibit another TCR in the same cell (70, 80–82).

Intriguingly, it has been shown that the tyrosine phosphatase SHP-1 is required for TCR antagonism (83) and two studies have documented that SHP-1 is recruited to the TCR after engagement of the TCR by antagonist ligands (81, 84); however, a direct role for phospho-ITAMs in SHP-1 recruitment was not shown. Instead, Stefanova, Altan-Bonnet and Germain proposed a model for TCR ligand discrimination where antagonist/weak agonist TCR/pMHC interactions result in recruitment of SHP-1 to TCR-associated LCK (84, 85). This interaction was inhibited by agonist TCR/pMHC interactions which resulted in (Ser59) modification of the LCK SH2 domain by activated pERK thereby blocking SHP-1 binding (84). However, a recent publication reported that a mutation of Ser59 to Ala in mice failed to yield the predicted loss of antagonism (86).

## A new *in vivo* mouse model of ζ ITAM function

**Figure 3:**
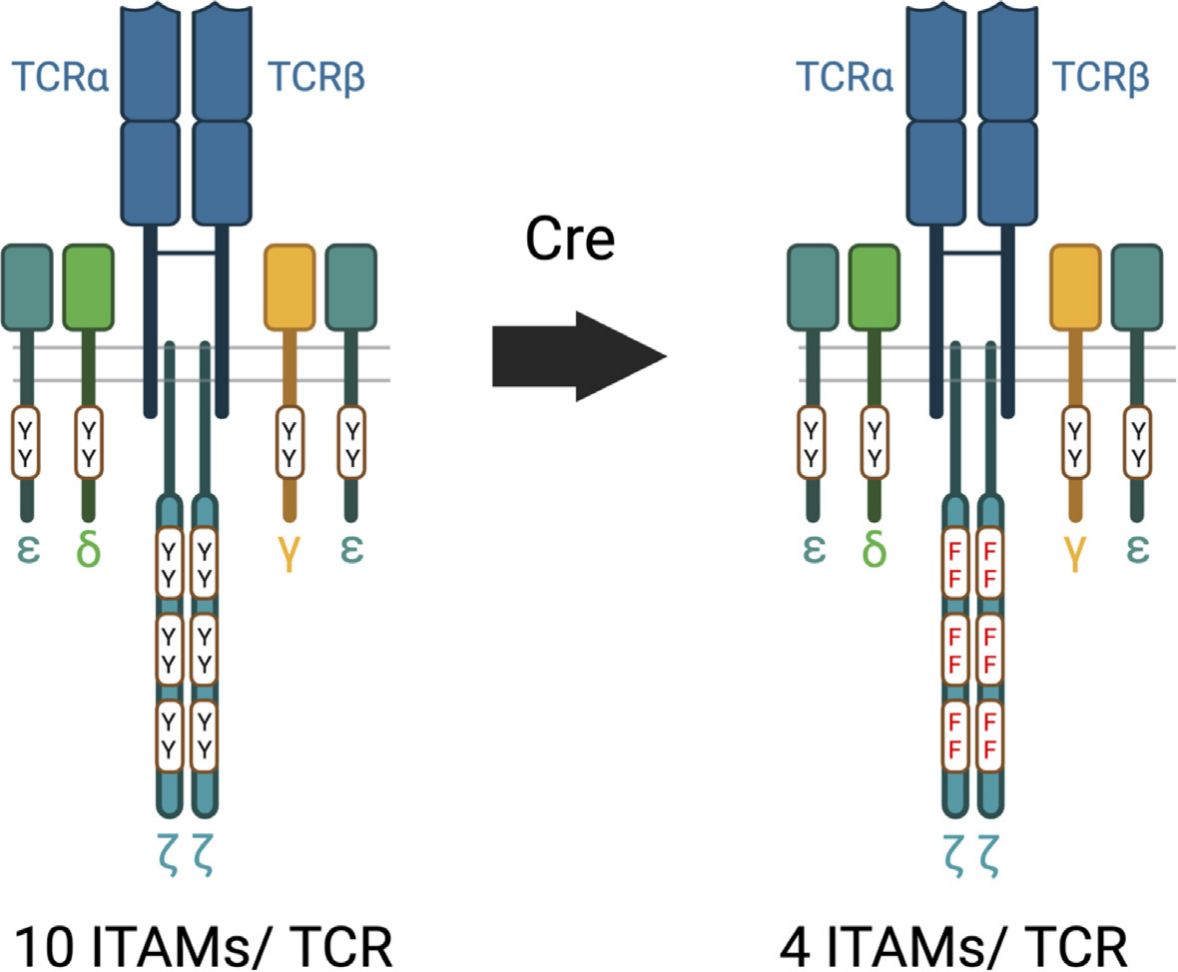
6Y and 6F TCRs. The wild-type 6Y TCR contains ten functional ITAMs within the CD3 and ζ subunits whereas the 6F TCR contains four functional ITMs within the four CD3 chains. Y=tyrosine; F=phenylalanine.

Several years ago, we became concerned that a problem with existing experimental models (including our own) used to assess the effects of ζ ITAM inactivation/deletion was that the timing and expression level of the transgenes or retro-transgenes used did not faithfully mirror that of endogenous ζ (45, 47, 57, 60, 64, 87). This was especially relevant for the analysis of key developmental stages and functional T cell responses that could be affected by developmental alterations caused by ζ ITAM inactivation. To address this issue, we generated an inducible ‘switch’ knock-in mouse model where a signaling defective ζ subunit with each of the six ITAM tyrosines mutated to phenylalanine (6F) ζ could be substituted for wild-type (6Y) after thymocyte selection and T cell maturation in the thymus (58, 59) (**Figure 3**). The 6Y/6F switch cassette was inserted into the endogenous ζ locus and phenotypic analysis confirmed that the timing and expression level of 6Yζ and 6Fζ matched that of endogenous ζ (58). Notably, analysis of germline 6Fζ mice revealed that the major steps of T cell development as well as the primary functions of T cells including proliferation and cytokine production were intact, confirming the remarkable results obtained with previous models that ζ mediated signals are dispensable for most general TCR controlled developmental and signaling responses. Post thymocyte development and selection switching of 6Fζ for 6Yζ using *dLck-Cre* or *ERT2-Cre* enabled the generation of mature, peripheral T cells that express 6Fζ but that had undergone development in the thymus using wild-type 6Yζ thus avoiding potential confounding problems from 6Fζ related developmental compensations (21).

## The 6Y/6F ζ-switch model reveals a dual function for ζ ITAMs that enables amplification, ligand discrimination and antagonism

**Figure 4:**
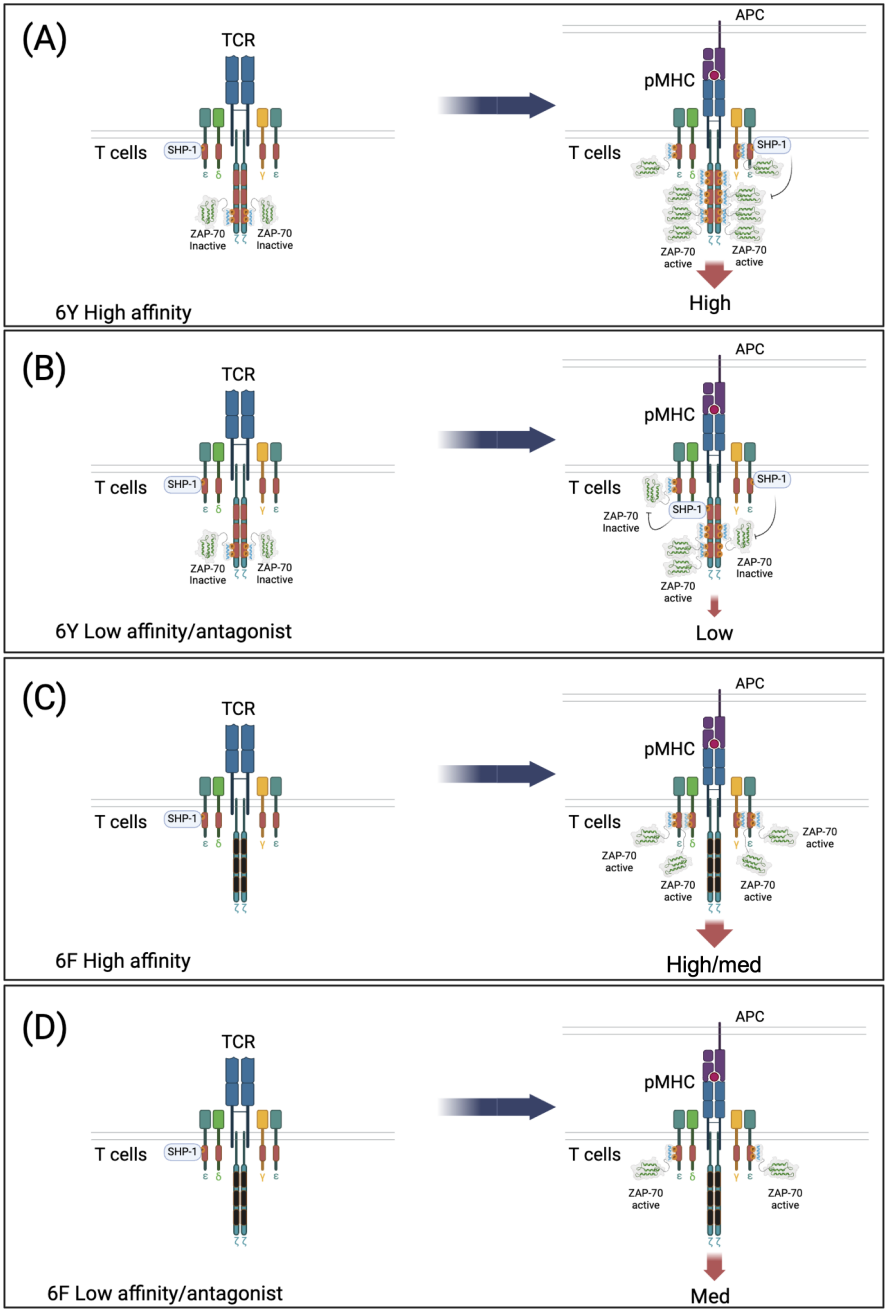
Model of ζ subunit dual function (amplification and inhibition) that is lost in 6F TCRs. 6Y TCRs recruit inactivated ZAP-70 in the resting state. Both 6Y TCRs and 6F TCRs can also recruit SHP-1 in the resting state. **(A)** Stimulation of 6Y TCRs with a high affinity ligand. Most or all of the ten ITAM tyrosines are di-phosphorylated resulting in abundant ZAP-70 recruitment and activation and transduction of a high intensity signal. **(B)** Stimulation of 6Y TCRs with a low affinity or antagonist ligand. Few ITAMs are di-phosphorylated and recruit ZAP-70, some ITAMs are mono-phosphorylated and recruit SHP-1 tyrosine phosphatase which limits ZAP-70 activation by de-phosphorylation of activating tyrosine(s) within ZAP-70 (and possibly dephosphorylation of ITAM tyrosines) resulting in transduction of a low intensity signal. **(C)** Stimulation of 6F TCRs with a high affinity ligand. All of the tyrosines within the four functional ITAMs are phosphorylated resulting in ZAP-70 recruitment and activation and transduction of a high/medium signal proportional to ITAM number. No ITAMs are mono-phosphorylated and SHP-1 is not recruited to/retained in the TCR. **(D)** Stimulation of 6F TCRs with a low affinity or antagonist ligand. Most of the four ITAMs are di-phosphorylated due to the reduced number of tyrosine targets of LCK resulting in ZAP-70 recruitment and activation. No ITAMs are mono-phosphorylated and SHP-1 is not recruited to the TCR, leading to the transduction of a medium intensity signal.

**Figure 5:**
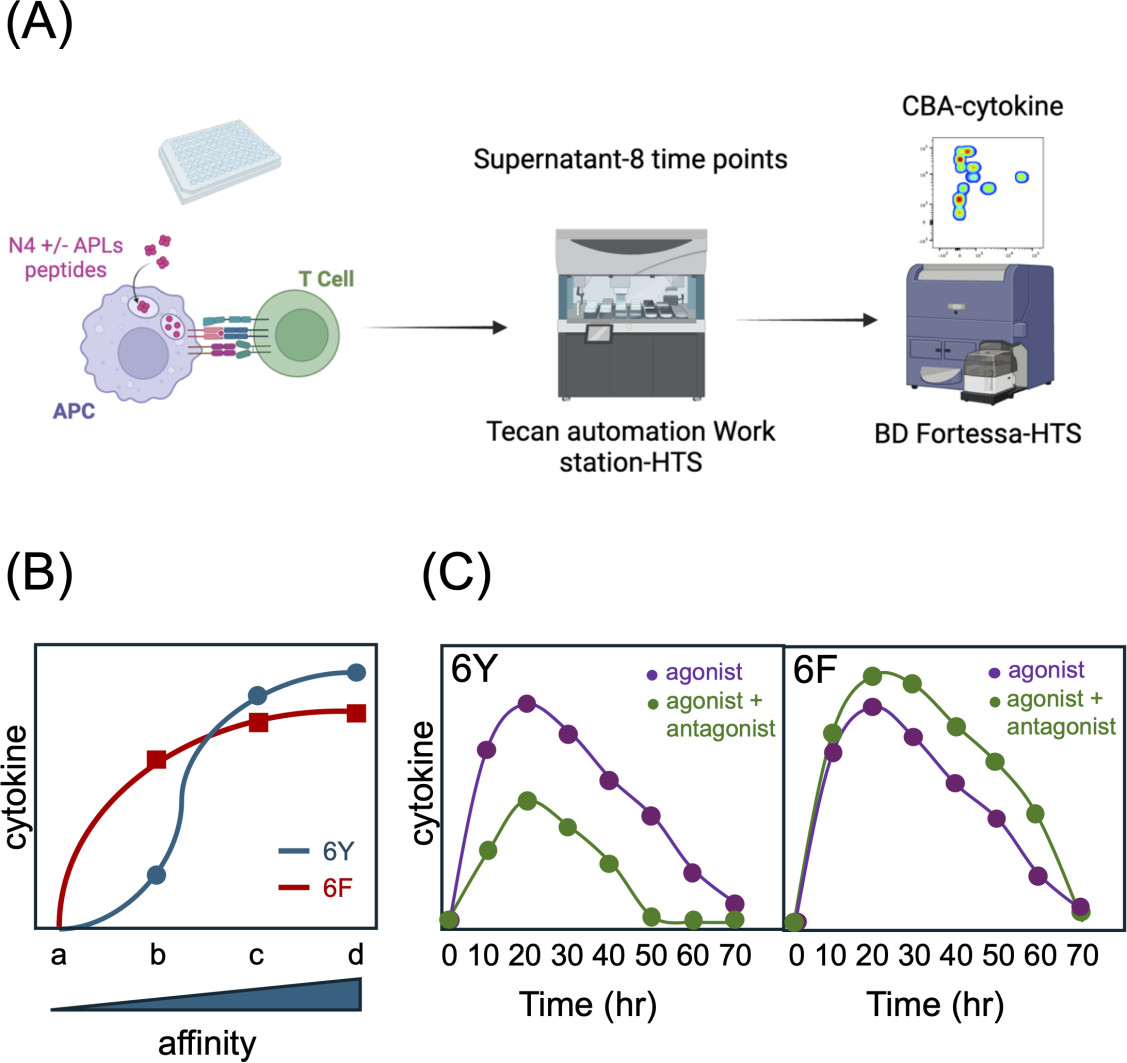
Effect of 6Fζ on TCR signaling responses. **(A)** Schematic of coculture experiments. **(B)** Stimulation of 6Y TCRs with antigens of increasing affinity for the TCR (a-d) results in a sigmoid cytokine output response (graph). 6F TCRs exhibit an enhanced response to low affinity ligands with an early plateau and a reduced response to high affinity ligands due to a reduction in total number of TCR ITAMs. **(C)** 6F TCRs are resistant to antagonism. The cytokine response of T cells expressing 6Y TCRs to agonist peptide is reduced in the presence of high concentrations of antagonist peptide whereas the response of T cells expressing 6F TCRs is not inhibited but instead enhanced in the presence of antagonist peptides which act as co-agonists. See Ref. 21 for more detail.

Using the 6Y/6Fζ mouse model, we were able to demonstrate that ζ has two functions in TCR signaling, activating or inhibitory, depending on the affinity of the TCR/pMHC interaction (21). High affinity (agonist) ligand interactions result in the phosphorylation of all six ζ ITAMs as well as all CD3 ITAMs which function additively to amplify TCR signals (**Figure 4A**). On the other hand, consistent with inhibitory ITAM models, low affinity TCR/pMHC interactions were shown to result in partial phosphorylation of ζ ITAMs and recruitment of SHP-1 phosphatase (**Figure 4B**). Importantly, this dual activating/inhibitory function of ζ ITAMs was lost in 6Fζ mice (**Figures 4C, D**) and was shown to be required for ligand discrimination (**Figures 5A, B**) and antagonism (**Figure 5C**) which were severely affected in 6Fζ T cells. Indeed, in most cases, peptides that acted as antagonists in 6Yζ T cells acted as agonists or partial agonists and weak agonists acted as moderate-strong agonists in 6Fζ T cells (**Figure 5C**). Although it remains uncertain whether the impaired feedback inhibition and antagonism seen in 6F-TCRs result from a unique regulatory function of ζ ITAMs or from a general reduction in the number of functional TCR ITAMs regardless of source within the TCR complex, these findings establish a direct connection between antagonism and ζ ITAM phosphorylation.

## Implications from the 6Y/6F ζ mouse model and remaining questions

Together with a large body of previous data from several laboratories, our new results obtained with 6Fζ mice suggest a model of TCR signaling in which CD3γ, δ and ε transduce signals that are sufficient for most or all TCR-mediated developmental and effector responses, and where ζ is included in the TCR primarily to enable ligand discrimination through its ability to transduce activating or inhibitory signals in a context (TCR-pMHC affinity) dependent fashion. Moreover, the mechanism employed by ζ to exert fine control of TCR-ligand responses, specifically recruitment of SHP-1 phosphatase, results, perhaps incidentally, in the phenomenon of antagonism. From this perspective, the unusual multi-subunit, multi-ITAM configuration of the TCR finally begins to make sense as it incorporates a mechanism that makes possible the exquisite sensitivity of the TCR, a property essential for thymocyte selection and ligand discrimination. We suggest that although ITAM multiplicity also facilitates signal amplification by high affinity ligand interactions, this property, often cited previously as the main role of ITAM multiplicity, is secondary. Unresolved questions that require additional investigation include whether the CD3γ, δ and ε ITAMs of the TCR also harbor regulatory (activating and inhibitory) activity, and the relevance (if any) of selective ZAP-70 recruitment to ζ ITAMs in response to homeostatic self-ligand TCR to inhibitory signaling by ζ.

This new model of ζ function could be used to further explore how TCR signaling operates in various contexts. From a basic science perspective, it would be interesting to investigate the impact of ζ ITAM variants in infection settings. One could speculate that enhancing the ability to respond to low-affinity antigens could expand the repertoire of reactive T cell clones. Additionally, examining how 6Fζ modified TCR signaling influences T cell exhaustion or clonal deletion will be of great interest.

As previously demonstrated, this new model of ζ function can also be leveraged to improve T cell based cancer therapies (21), particularly given that most tumor neoantigens are of low affinity and their capacity for activating T cells should be increased by expression of 6Fζ (88). One potential approach would involve transducing bulk Tumor-Infiltrating Lymphocytes (TILs) from patients and assessing whether the modified TILs exhibit enhanced cytolytic activity against the autologous tumor. Another current immunotherapy strategy involves expressing cloned tumor-specific TCRs in autologous peripheral T cells before reinfusing the redirected T cells into the patient. While this approach holds promise, only high-affinity TCRs have been employed successfully to date. Co-expression of 6Fζ could enhance the TCR signaling response of low affinity TCRs (and possibly also convert antagonist TCR responses to agonist TCR responses), enabling the use of abundant but under-utilized tumor-specific TCRs and broadening the pool of effective neo-antigen specific TCRs.

In conclusion, new data indicate that the singular multi-subunit/multi-ITAM configuration of the TCR enables graded signaling responses to ligands of different affinity enabling TCR ligand discrimination. If the lessons from the past three decades are heeded, continued TCR structure/function studies should be well worth the effort, as it is altogether likely that the TCR still holds secrets yet to be discovered.
